# Insights in Behavior of Variably Formulated Alginate-Based Microcapsules for Cell Transplantation

**DOI:** 10.1155/2015/965804

**Published:** 2015-05-20

**Authors:** Pia Montanucci, Silvia Terenzi, Claudio Santi, Ilaria Pennoni, Vittorio Bini, Teresa Pescara, Giuseppe Basta, Riccardo Calafiore

**Affiliations:** ^1^Interdisciplinary Laboratory for Endocrine Cell Transplants and Biohybrid Organs, Department of Medicine, Section of Internal Medicine and Endocrine and Metabolic Sciences, University of Perugia, Via Enrico dal Pozzo, s.n.c., 06126 Perugia, Italy; ^2^Department of Pharmaceutical Sciences, University of Perugia, Via del Liceo 1, 06164 Perugia, Italy; ^3^Department of Medicine, Section of Internal Medicine and Endocrine and Metabolic Sciences, University of Perugia, Via Enrico dal Pozzo, s.n.c., 06126 Perugia, Italy

## Abstract

Alginate-based microencapsulation of live cells may offer the opportunity to treat chronic and degenerative disorders. So far, a thorough assessment of physical-chemical behavior of alginate-based microbeads remains cloudy. A disputed issue is which divalent cation to choose for a high performing alginate gelling process. Having selected, in our system, high mannuronic (M) enriched alginates, we studied different gelling cations and their combinations to determine their eventual influence on physical-chemical properties of the final microcapsules preparation, *in vitro* and *in vivo*. We have shown that used of ultrapure alginate allows for high biocompatibility of the formed microcapsules, regardless of gelation agents, while use of different gelling cations is associated with corresponding variable effects on the capsules' basic architecture, as originally reported in this work. However, only the final application which the capsules are destined to will ultimately guide the selection of the ideal, specific gelling divalent cations, since in principle there are no capsules that are better than others.

## 1. Introduction

Alginic acid, a polysaccharide originally extracted from brown seaweeds, and its salts have historically represented the most common material to fabricate microcapsules used to envelop, mainly, although not solely, pancreatic islet cells. Alginates are linear copolymers composed of two building units, *β*-D-mannuronic (M) and *α*-L-guluronic (G) acids, mainly patterned through the entire molecule in the form of MM or GG or MG dimeric blocks. The most relevant characteristic of the alginates is the selective binding to multivalent cations, a property that allows for formation of alginate gel beads [1]. The gel formation always implies a process of specific ion exchange. The starting point is a water soluble alginate salt made of monovalent cations like sodium or potassium as counterions which have to be exchanged with divalent cations in order to activate gelling by a monocation displacement process. The affinity of alginates for divalent cations depends on their composition [2–5]. Guluronic acid-based alginate is more prone to ion binding compared to the mannuronic acid-based product, while the affinity for the alkaline earth metals changes by the following order: Mg ≪ Ca < Sr < Ba [1, 5].

The divalent metals (i.e., Cu, Cd, Ba, Sr, Ca, Zn, and Co) diffuse into an alginate solution and the cation-binding crosslinks the alginate in a highly cooperative manner, thereby forming a gel, with the crosslinking density being based on the original ion concentration. It has been described that Ca^2+^ cations bind both to G sequences and to alternating GM dimeric blocks but not to M-blocks only; Sr^2+^ ions bind well to polyG and not at all to polyM, while a very limited binding is detected for polyMG; Ba^2+^ ions bind to separate M and G blocks but not to hybrid MG sequences [6].

The high selectivity between similar ions, such as alkaline earth metals, indicates that the binding mode cannot be simply related to nonspecific electrostatic interactions but reasonably involves a precise chelation process that mainly depends on the chemical distribution of the G blocks. This property has been explained with the egg-box model [7, 8] based on the steric configuration of the G blocks residues. This model explains the gel formation through the displacement of Na^+^ by Ca^2+^ ions from two adjacent G blocks to form a single ion bridge between the alginate's chains. All this suggests a cooperative binding mechanism between two or more chains: while the Ca^2+^ ions help hold together the alginate chains, their polymeric nature leads them to bind to calcium in a more stable fashion. Structure of the G chains provides the correct distance for a high degree of coordination of calcium ions between the carboxyl and hydroxyl groups [7]. The theoretical explanation for this behavior is based on a self-cooperative process between neighboring elements (Ising model) and is based on a physical bond with unfavorable entropy for the first divalent ion. The bond is favored for all ions so as to form a one-dimensional egg-box (zipping mechanism). Gelling kinetics is fast and adapts to an entrapment process where a single alginate drop turns into a single gel bead incorporating cells or drugs of various natures [9]. Using a special microdroplet generator, microcapsules measuring an average of 300–800 *μ*m in diameter can be smoothly prepared [10].

In the field of microencapsulated live cell/tissue transplantation, the most widely used gelling cation has been calcium owing to its chemical versatility and safety [10–13]. However, others have employed other cations such as barium [14, 15]: this has been often preferred to calcium since it forms more resistant gels [4, 16] and simplifies the capsules' chemistry by omitting an otherwise necessary aminoacidic polycation coating [10]. Nevertheless, barium toxicity is well known and this fueled worries with regard to* in vivo* use of Ba-alginate microcapsules. However, barium release from G-enriched alginate has been proven to lag much below the toxicity threshold [17]. Others emphasized that the Ba-related gel strengthening effects are associated only with G block concentrations exceeding 60% [6], and low barium should also be advantageously added as a companion cation to calcium (Ba^2+^ 1 mM, Ca^2+^ 50 mM) [14]. Alginates virtually represent the only materials associated with good biocompatibility and favorable porosity/permeability properties, which have, so far, fulfilled criteria for human application, provided that they undergo adequate purification. Purification is necessary because they are contaminated by high endotoxin levels, pyrogens, proteins, and heavy metals [18].

Microcapsules made by ultrapurified, “clinical-grade” alginates, as devised by our laboratory, usually do not provoke any inflammatory cell reaction, as extensively proven by our comprehensive* in vivo* studies [19, 20]. Due to this relevant preclinical background, the Italian Institute of Health, in compliance with regulations of the European Medicine Agency (EMA) and the US Food and Drug Administration (FDA), granted us permission to initiate a closed pilot clinical trial of microencapsulated human islet transplantation into nonimmunosuppressed patients with T1D [21, 22]. Purpose of this work was then to meticulously determine* in vitro* long-term stability and* in vivo* biocompatibility of microcapsules made of the ultrapure high-M alginate made with different divalent gelling cations in order to provide critical and innovative information with regard to transplant application of encapsulated cells.

## 2. Materials and Methods

### 2.1. Alginate Characteristics

Powdered alginate was purchased from Monsanto-Kelco featuring the following properties: molecular weight = 120,000–190,000 kDa; mannuronic acid (M) and guluronic acid (G) = M fraction (*F*_M_) 61%; G fraction (*F*_G_) 39%. It is a “high-M” alginate.

Alginate ultrapurification was conducted under GLP conditions, based on patent number WO 2009093184 A1. At the end of the process, the obtained alginate solution properties were the following: (1) endotoxin level, measured by LAL test, <27.8 EU/g (<0.5 EU/mL) (any level below 100 EU/g in this test is considered endotoxin-free), (2) protein content <0.45%, (3) viscosity 100–300 cps, (4) heavy metal content below the recommended cut-off, and in particular, Ca<100 ppm; Cu<40 ppm; Fe<60 ppm; Hg<40 ppb; Mg<40 ppm; Zn<40 ppm; Pb<50 ppm; Si<10 ppm; Mn<10 ppm; Sr<40 ppm; As<100 ppb.

### 2.2. Preparation of Alginate Microcapsules

Microcapsules were prepared, according to our SOPs, starting from 1.8% high-M sodium alginate solution, produced as previously described [10] with the exception of the outer poly-L-ornithine (PLO) coating that was omitted. The same physical-chemical parameters were used for all experiments. Briefly, the alginate solution was continuously aspirated, at a fixed flow rate, by a peristaltic pump and extruded through a microdroplet generator; the resulting microdroplets were collected into solutions containing the examined divalent cations which immediately made them turn into gel microbeads. The employed gelling solutions were 100 mM CaCl_2_, 50 mM BaCl_2_·2H_2_O, 50 mM CaCl_2_, and 25 mM BaCl_2_·2H_2_O, 100 mM SrCl_2_·6H_2_O, with these salts (Sigma-Aldrich) being dissolved in sterile NaCl 0,9%. All the experiments were conducted on all types of microcapsules, gelled by Ca^2+^, Ba^2+^, Ca^2+^-Ba^2+^, and Sr^2+^. After the gelling, the microcapsules were retrieved, washed twice in saline, and maintained at +4°C in the same buffer for further examination. Characterization of the microcapsules was performed O/N under light microscopy assessment (using Nikon Eclipse TS100 microscope). In particular, capsules' integrity and average size were determined.

For* in vivo* testing upon their production, the microcapsules in complete culture medium were incubated for additional 24 h at 37°C 95% air/CO_2_ for sterility evaluation. The microcapsules were then divided into three equivalent groups: one aliquot was maintained at +4°C in saline while the other two aliquots were used for intraperitoneal graft in NOD/SCID and CD1 mice, respectively.

### 2.3. *In Vitro* Studies

It is known that Na^+^ contained in the 0.9% sodium chloride solution can compete with the capsules' gelling cations and alter the capsular texture [6]. In particular, the microcapsules can swell, becoming more porous or even burst. For this reason, stability studies have been performed by measuring the capsules' equatorial size over time under different conditions (for each test a minimum of 20 measurements were performed). On this purpose, 1 mL of microcapsules upon gelling by different cations were placed in a 6 multiwell in 3 mL of saline at 4°C. The capsules' integrity and size were evaluated at 10 days, 1 month, 2 months, 4 months, 5 months, 6 months, and 7 months of the fabrication and compared with the assessment made at the beginning.

In order to determine if the microcapsules have released ions, the saline solution, in which the microcapsules were stored for 7 months, was also analyzed for sodium, barium, calcium, and strontium content in comparison to fresh saline solution.

The influence of the temperature on the microcapsules' stability also was evaluated. 1 mL of freshly prepared microcapsules was placed into two 6 multiwell plates, one at 4°C and the other one at 37°C. The microcapsules' stability was again examined after 4, 6, 8, and 10 days and finally 2 months from production and compared with the starting values.

The microcapsules' diameters were also measured as “resistance against osmotic swelling” in isotonic saline solution, after repeated saline changes. An aliquot (100 *μ*L) of freshly prepared and stored O/N in gelling solution, or 7-month-old microcapsules, in 2 mL of saline, was transferred into a 6 multiwell plate in 3 mL of saline. The solution was changed and the incubation went on for 1 hour. Afterwards, capsules' diameters were measured. This procedure was repeated and the particle size was measured again after O/N and after 1 hour. This test was performed also at +4°C. At the end of the procedure, the saline solution withdrawn from the last change was analyzed for ions content as mentioned below.

To better understand the nature of the phenomenon driving microcapsule diameters' variations, we set up additional experiments.

In particular, we have examined the resistance to mechanical agitation; to this end, 100 *μ*L of microcapsules in 2 mL of saline in 6 multiwell plate was placed on an orbital shaker set at 140 rpm at +4°C. Assessment of the integrity and size of the capsules was conducted at 1 hour, 16 hours, and 24 hours.

We also performed a “fake saline change” on an aliquot (100 *μ*L) of microcapsules standing in 2 mL of saline solution at +4°C for 7 months into a 6 multiwell plate: the aspirated saline was transferred back to the plate with no changes. We here aimed at disturbing the solution equilibrium with regard to ions' concentration and distribution around the capsules (a steady state level is accomplished by the time). After 1 hour of change, we have measured the size of the microcapsules, with the procedure being repeated twice. At the end, we collected the salines to analyze the content of sodium, barium, calcium, and strontium in comparison with fresh saline as mentioned below. As an additional experiment, after assaying the counterions' concentration in the saline containing 7-month-old microcapsules, capsules aliquots were placed in fresh saline, to which counterions were supplemented so as to obtain the saline assayed ion concentration as reported below.

### 2.4. Assay of Barium, Calcium, Strontium, and Sodium in Saline

Ionic content (Ba^2+^, Ca^2+^, Sr^2+^, Na^+^) in the various saline solutions was determined by inductively coupled plasma-optical emission spectroscopy (ICP-OES, 720-ES Varian equipped with the autosampler SP3, Varian). All the samples were diluted 10 times with deionized water. Concentrated nitric acid was also added to a final 5% (v/v) concentration. Measurements were done using a concentric sea-spray nebulizer and a cyclonic double pass spray chamber. The assays included the use of yttrium (1 mg/L) as an internal standard with CsNO_3_ 1 g/L as an ion buffer. The internal standard solution was added on line using a Y connection. The ion concentrations were quantified using an external calibration (0.01/0.1/1/10 mg/L) and a quadratic fitted line.

### 2.5. *In Vivo* Stability Studies (NOD-SCID Mice)

NOD/SCID mice (*n*° = 8) were divided into four groups, of two mice each, and transplanted with either Ca^2+^-alginate beads, Ba^2+^-alginate beads, Ca^2+^-Ba^2+^-alginate beads, or, finally, Sr^2+^-alginate beads. We selected, in a preliminary study phase, such rodent animal models to assess the stability of the capsules over time, with no immune system-related interferences. All mice underwent general anesthesia (0,01 mL/g Zoletil-Rompum), with 1 mL of microcapsules being suspended in 2 mL of sterile saline before intraperitoneal transplantation (TX). At 40 days of TX, the capsules were retrieved by peritoneal lavage, examined under light microscopy and by NMR analysis, and compared with the original capsules batch, maintained* in vitro* at +4°C.

### 2.6. *In Vivo* Biocompatibility Studies (CD1 Mice)

We then used an immunocompetent mouse strain (CD1) to evaluate the biocompatibility of the capsules gelled with the different cations. In particular, 8 CD1 mice were divided into four groups, of two mice each, transplanted with either Ca^2+^-microcapsules, Ba^2+^-microcapsules, Ca^2+^/Ba^2+^-microcapsules, or, finally, Sr^2+^-microcapsules, respectively. All mice were implanted as previously described. One mouse per group was sacrificed 40 days after transplantation, while the remaining mice were injected intraperitoneally with 2 mL LPS (Lipopolysaccharide prepared at 0,1 mg/mL in sterile saline, from Sigma-Aldrich) to induce a strong chemical peritoneal inflammation. Six days after LPS inoculation, the mice were sacrificed, with the microcapsules being recovered by accurate peritoneal lavages. All the explanted microcapsules were examined under light microscopy in order to detect any eventual biological response elicited by the grafts and subsequently analysed by NMR in comparison with the same capsule batches maintained* in vitro* at +4°C.

All the treated mice were cared for following the animal welfare guidelines adopted by the University of Perugia. All the experimental procedures involving animals were approved by the local ethical committee.

### 2.7. Preparation of Sodium Alginate Samples Derived from Microcapsules for NMR

To perform the NMR analysis, the capsules maintained* in vitro* and those recovered from the transplanted mice were dissolved using Na-EDTA (100 mM in 0.9% NaCl pH = 8) added to the capsules according to the proportion of 800 *μ*L of solution/250 *μ*L of capsules [23]. After lyophilization (24–36 hrs), the samples without further purification were dissolved in deuterated water and were analyzed by NMR using the conditions optimized for the native 1.8% Na alginate.

### 2.8. NMR Analysis

We recently reported a protocol for the NMR analysis of nonhydrolysed samples of sodium alginate in D_2_O [18]. Low viscosity solutions can be obtained, affecting the experiments at 338 K. At this temperature, the direct acquisition of well-resolved spectra avoiding the acidic pretreatment of the alginate at 373 K for 1 to 3 hours was performed. In addition, the heating during the NMR examination moved the HOD signal to high field resonances, far away from the diagnostic frequencies of the anomeric proton of the polymer.

20 mg of solid sodium alginate (or of the lyophilized degelling mixtures) was dissolved in 1 mL of D_2_O and analyzed in a Bruker NMR Avance 400 MHz instrument. The spectra were recorded without the suppression of the water and the signals were assigned on the basis of the data previously reported in the literature and confirmed on the base of 2D-COSY and NOESY correlations [24–26]. From the integrals of the peaks, it is possible to estimate both the ratio mannuronic (M) and guluronic (G) acidic residues, along the polymer chains, and the frequencies of occurrence of diad uronic acid residue pairs as molar fraction of the polymer.

From the comparison between these spectra and those obtained from hydrolyzed samples of sodium alginate, it was possible also to assign the signals of the anomeric protons of the reducing end-groups (signals in the range of 5.15–5.10 ppm M*α* and G*α* and broad signals in the range of 4.86–6.76 ppm M*β* and G*β*) [27].

From the evaluation of the ratio between the integrals relative to these signals and those of the polymer, it is possible to estimate the grade of hydrolytic depolymerization and, consequently, the stability of the polymer when it was subjected to different treatments [18].

It has been reported that the affinity of different crosslinking ions for the alginate strictly depends on its composition and reflects the ability of the cation to coordinate poly-G, poly-M, or alternate sequences [6]. The analysis of calcium alginate in the condition optimized for the sodium derivative did not produce interpretable NMR spectra because of the high viscosity of the gel. However, after ion exchange degelling obtained by treatment of the alginate gels with Na-EDTA, it is possible to affect the analysis of the resulting material and evaluate the composition of the polymer sequences after the selection of the crosslinking agent from the sodium native matrix. Using this procedure, we comparatively examined the polymer composition of the capsules prepared by gelling the alginate with calcium, barium, and strontium as well as with a mixture of calcium and barium. In all the cases, the signals of the impurity derived by the different treatments of the capsules and by the degelling did not affect the anomeric signals and, in comparison with the spectra of the native sodium alginate, only a moderate general broadness was observed, indicating a different viscosity of the sample.

### 2.9. Statistical Analysis

Distributions of variables were assessed by Shapiro-Wilk test and data were standardized, for each ion, on mean and SD of baseline diameters (*z*-score). Two-way analysis of variance for repeat measures (RM ANOVA) was used to detect differences among the groups; then “time” (day 0–210) and “group” (kind of ion) were considered as “within-subjects” and “between-subjects” factors with four “time” and four “group” levels, respectively. Polynomial contrasts for trends and post hoc tests were additionally performed; in this instance, a Bonferroni correction was applied in order to take into account multiple comparisons. For “within-subjects” effects the Mauchly criterion was used to determine if the assumption of sphericity was met.

Statistical analyses were performed using IBM-SPSS version 21.0 (IBM Corp., Armonk, NY, USA, 2011). A two-tailed *P* value < 0.05 was considered significant.

## 3. Results

### 3.1. Production of Alginate Microcapsules

**Figure 1:**
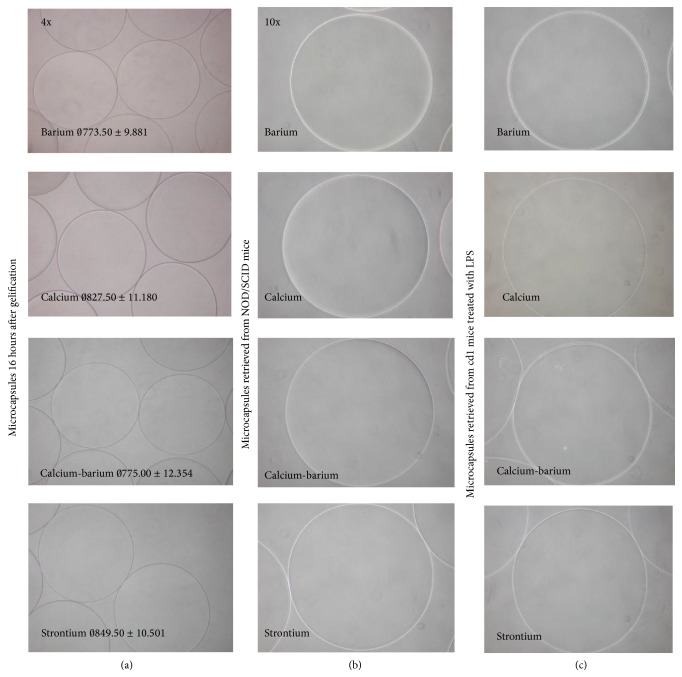
Microcapsules' integrity and morphological evaluation. (a) Microcapsules 24 hours after gelification with 100 mM CaCl_2_, 50 mM BaCl_2_·2H_2_O, 50 mM CaCl_2_, and 25 mM BaCl_2_·2H_2_O, or 100 mM SrCl_2_·6H_2_O in 0.9% NaCl. All types of microcapsules show a spherical and smooth shape and are homogeneous in diameters' size for each type of counterion used. (b) Microcapsules retrieved from Nod/Scid mice after 40 days from transplantation retained their physical-chemical parameters, with no change in terms of morphology. (c) Microcapsules retrieved from CD1 mice after 40 days from transplantation and 6 days with LPS: they appear undamaged and free from any inflammatory response with no macrophage growth outside the membrane.

We observed that the different gelling agents were associated with various effects on the capsules diameter: using barium or the calcium-barium ions, the average diameter was similar; calcium only yielded larger capsules, although these were the largest with strontium (Figure 1(a)).

### 3.2. *In Vitro* Capsules Evaluation

**Figure 2:**
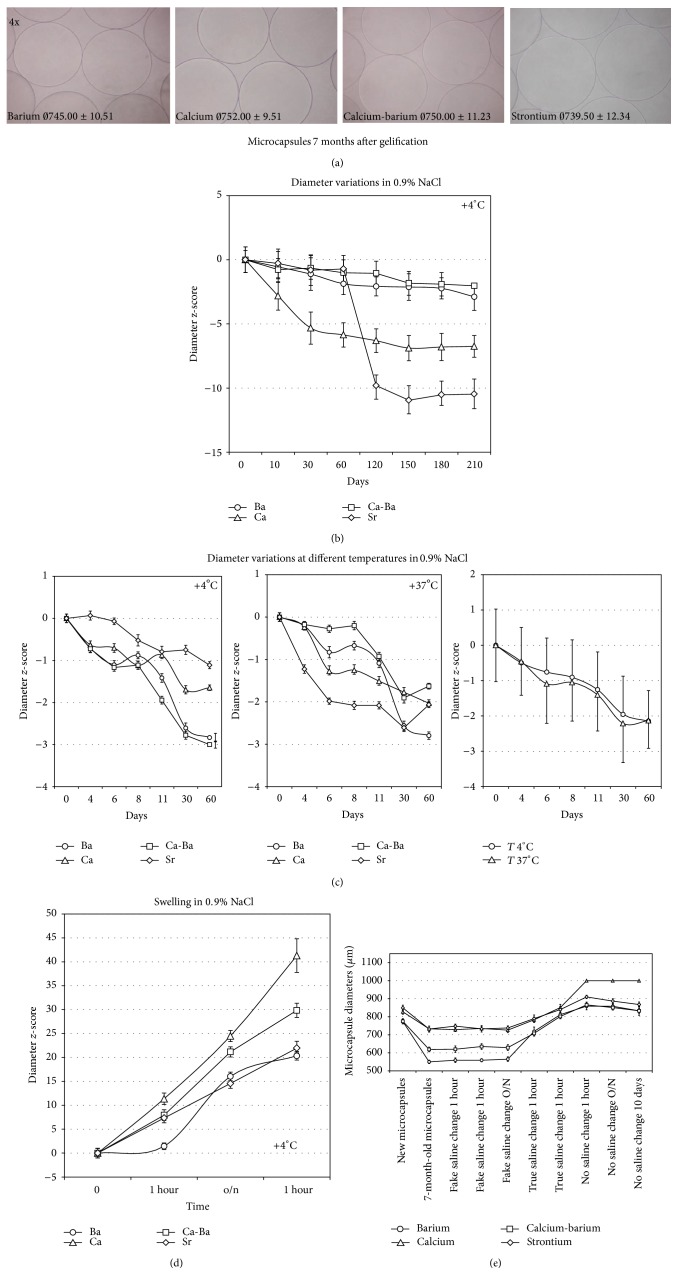
*In vitro* evaluation of microcapsules. (a) The microcapsules gelled with indicated conterions 7 months after gelification and maintained in saline at +4°C maintain a spherical and smooth shape. (b) Distribution of diameters' size over the time for each microcapsule's type at +4°C. All microcapsules show the aptitude to decrement their diameter both over time (*P* < 0.0001) and between ions (*P* < 0.0001). (c) The diameter sizes show similar differences over time (*P* < 0.001) and between ions (*P* < 0.0001) in both left (+4°C) and middle (+37°C) panels; the temperature is irrelevant on diameters' decrement for microcapsules made with various gelling solutions, as shown in the right panel in which, considering all the ions together, the within-subjects effect (time) is still significant (*P* < 0.0001), whilst the between-subjects effect (temperature) is not significant (*P* = 0.122). (d) Microcapsules left O/N in gelling solution swell when saline solution was changed as indicated in figure both over time (*P* < 0.0001) and between ions (*P* < 0.0001). (e) The illustrated graph summarizes at once, using the same capsules, some of the experiments carried out throughout this report. The initial part of the graph shows capsules maintained in saline for 7 months. The second part shows the experiment elsewhere named “fake saline changes,” while the last part coincides with the swelling test performed by changing saline at fixed times. Finally, the capsules have been left to rest in saline, like the beginning of the experiment. Looking at the graph, during the “fake saline changes” the capsules' diameters did not vary compared to the values seen after 7 months of incubation. Two actual changes of saline were performed; all capsules' types tended to increase their diameter. The capsules then were incubated for 1 hour at 4°C, overnight, or for 10 days with no saline changes. From the graph, it appears that the capsules tend to stabilize their diameters which eventually tend to decrease again.

All the microcapsules preserved sphericity and exhibited smooth surface by the time of preparation; these properties were uniquely retained at seven months of storage at 4°C in 0.9% NaCl (Figures 1(a) and 2(a)). In fact, the microcapsules after 7 months of storage in saline did not exhibit “swelling” and no broken capsules were found. Throughout* in vitro* maintenance, all microcapsules tend to reduce their size (Figure 2(b)). In particular, statistics of diameter assessments showed that elapsing time was significantly associated with capsule's size reduction. Moreover, statistical analysis indicates that (data not shown) reduction is not only related to time but also related to the selected gelling ion.

In principle, every ion of the gelling solution induces time-related capsule's size reduction but on a variable manner. Trend statistics show that diameter of capsules is associated with both linear and quadratic trends (*P* < 0.0001). Interaction time and type of used ion in combination are also significant and different between the various capsule types (*P* < 0.0001). Within such a distribution, capsules' diameter tended to decrease over time. Ba- and Ca-gelled capsules behead quite similarly, as far as time-related diameter reduction was concerned. The only exception was strontium, as gelling agent, since it induced relative capsules shrinking, between 60 and 120 days, and diameter decline from 842.00 ± 11.05 *μ*m to 746.50 ± 8.75 *μ*m, followed by final stabilization.

Capsules' diameter measurement, after storage at +4°C or 37°C for 60 days, showed that temperature did not seem to influence capsules' morphologic integrity and diameter. In fact, all types of microcapsules showed the same diameter reduction, as described above (Figure 2(c)).

Since all the microcapsules, if not manipulated, regardless of the gelling ions, tend to shrink over time, we performed “ad hoc” experiments in order to interpret this phenomenon. In fact, we would expect an increase of diameter (swelling) over time since the microcapsules were stored in 0.9% NaCl solution. These experiments included freshly prepared or 7-month-old microcapsules and the evaluated tests were resistant to osmotic swelling in isotonic saline, to strong mechanical stress, or to equilibrium disruption by “fake saline changes.”

In particular, either fresh or long-term stored (7 months) capsules showed pronounced swelling upon the first saline change. This trend faded away upon the second and the third change. Only those microcapsules that were left O/N in gelling solution [6] looked more resistant to swelling, still increasing in diameter (Figure 2(d)). More sensitive to swelling were those capsules fabricated with Ca^2+^ as gelling ion. Ca-based capsules showed an average diameter of 594.50 ± 9.99 *μ*m at the beginning and 1007.00 ± 35.01 *μ*m at the end; Sr-based capsules showed an average diameter of 648.50 ± 12.70 *μ*m at the beginning and 927.00 ± 18.09 *μ*m at the end. Only those capsules that were gelled with Ba^2+^ or Ca^2+^/Ba^2+^ did not overcome 900 *μ*m in diameter (Ba^2+^ from 581.50 ± 114.96 *μ*m to 886.00 ± 13.14 *μ*m; Ca^2+^/Ba^2+^ from 585.50 ± 9.44 *μ*m to 867 ± 14.09 *μ*m). This swelling phenomenon is what we expected from our microbeads after some months of storage in saline solution.

Why do the diameters shrink over time when the microcapsules were kept in saline solution for 7 months? Mechanical stress is not the answer because our 7-month-old microcapsules, exposed to strong mechanical stress (orbital shaker), showed no change. Also the “fake saline change” and the average size of microcapsules' diameters were comparable to those measured at the beginning of the experiment (Figure 2(e)). By this experiment, we wanted to exclude that a simple physical phenomenon (counterions around the beads) prevents the sodium ions' penetration into the microcapsules making them swell. Since the result of the “fake saline change” was negative (no swell after pipetting), it is likely that the ion composition of the saline solution accounted for the swelling phenomenon. For this reason, we collected the saline solutions and analyzed their ion content in comparison to fresh saline solutions. In Table 1, the ions present in the supernatants of the capsules stored in saline after 7 months at 4°C, from “fake saline change” and “after swelling” of fresh or old capsules conditions, were examined. Sodium concentration was unchanged in all the solutions. The Ba^2+^, Ca^2+^, and Sr^2+^ concentrations were practically the same in the saline solution of 7-month-old microcapsules (with or without “fake saline change”). At the same time, we observed that the ion concentration of the fresh saline solution after 2 or 3 changes contained almost 1/3 of Ba^2+^, Ca^2+^, and Sr^2+^ compared to the the saline solution of 7-month-old beads. Since we knew the ions concentration of the saline solution after 7 months, we tried a new experiment. We changed the saline solution of the 7-month-old microcapsules for a fresh saline solution spiked with ions at the same concentration found after 7 months. In this case, the microcapsules of Ba^2+^ and Ca^2+^- Ba^2+^ are unchanged, whereas those made with Ca^2+^ or Sr^2+^ swell slightly; upon a small initial swelling, the microcapsules returned to their initial diameter (after 2 hours) (data not shown). Probably, after a certain time an equilibrium between the saline solution and the intracapsular composition was reached, which stabilized the microcapsules' size.

### 3.3. *In Vivo* Stability Studies (NOD-SCID Mice)

Twenty-eight days after transplantation into eight NOD-SCID mice, the microcapsules were explanted. The microcapsules looked free of inflammation and were freely floating in the peritoneum, with no adhesion to the intra-abdominal organs (Figure 1(b)). Upon accurate washing, so as to discard blood or peritoneal cells, the capsules were resuspended in sterile poly-saline (i.e., Krebs's solution) and maintained at +4°C for examination under light microscopy analysis and, after degelling and lyophilization, by NMR analysis. The microcapsules gelled with the different cations did not show any significant difference: they retained their physical-chemical properties during transplantation, with no morphological changes.

### 3.4. *In Vivo* Biocompatibility Studies (CD1 Mice)

All microcapsules were retrieved and found free of inflammatory response and pericapsular tissue overgrowth. Hence, alginate microcapsules did not induce any immune response by the host. These results confirmed high biocompatibility of the purified alginate, regardless of the used gelling cations. Also barium, potentially associated with intrinsic toxicity, did not induce any acute response. Moreover, microcapsules remained stable, confirming findings of previous studies in NOD-SCID mice. Microcapsules were not affected by inflammation caused by LPS that was injected intraperitoneally. In fact, all the retrieved microcapsules were free of any inflammatory response and remained stable. Hence, the artificially induced inflamed environment did not cause any degradation process of the alginate microcapsules and did not affect microcapsules' features, such as morphology (Figure 1(c)).

### 3.5. NMR

**Figure 3:**
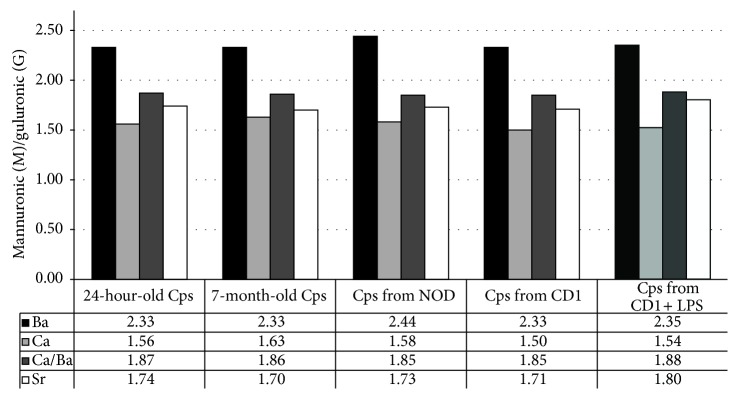
Evaluation of mannuronic (M): guluronic (G) acidic residues ratio.

NMR analysis was effected on samples of sodium alginate obtained by the above described degelling procedures from the microcapsules standing* in vitro* for 24 hours or 7 months, and on microcapsules retrieved from NOD/SCID, CD1, and CD1 mice after LPS inoculation. In all the analyzed samples, the material obtained by degelling showed almost the same M/G composition depending on the cation originally used for the crosslink gelling (Ca = 1.50–1.63; Sr = 1.70–1.80; Ba = 2.33–2.44; Ca-Ba = 1.85–1.88) with no evidence of hydrolytic depolymerization being observed (Figure 3).

From the integrals of the peaks on NMR analysis, it is possible to estimate the ratio mannuronic (M): guluronic (G) acidic residues, along the polymer chains. From our analysis, it is possible to assume that there are no differences in M/G ratio depending on the cation originally used for the crosslinking gelification between all shown conditions.

## 4. Discussion

Size of the alginate-based (high-M) microcapsules, regardless of shape and smoothness that remained anyway constant, was shown to significantly depend upon the nature of the employed gelling cation. When gelling was based on Ba^2+^ or Ca^2+^-Ba^2+^, the process is driven by barium. In fact, owing to its high ionic radius, it is difficult for Ba^2+^ to form “egg boxes” that require GG sequences. For this reason, Ba^2+^ may select alginate patterns where the M/G ratio is higher (from NMR data). The initial diameter of the Ca- was higher than Ba-gelled capsules, likely indicating the scarcity of egg-box reacting GG sequences that are typical of high-M patterned alginates; Sr-gelled capsules were larger for the same reason. NMR analysis of the degelled capsules confirms that the mannuronic/guluronic (M/G) ratio directly depends on the ionic radius of the gelling cation and it matches the affinity previously reported [6] for barium, calcium, and strontium with GG, MM, and MG sequences. Staying with the sodium alginate used in the present work, the contribution of MG and MM block cannot be overseen. In particular, we must consider that Ca^2+^ ions bind both to G and to MG dimeric blocks but not to M blocks, while Ba^2+^ ions bind both to the M and to the G blocks, apparently regardless of the MG block sequences. The results obtained with strontium, in our opinion, are the consequence of the limited MG affinity of this crosslinking agent that showed higher affinity for the polyG than for the polyM. Interestingly, when sodium alginate was gelled with a mixture of calcium and barium, an intermediate M/G ratio was obtained demonstrating that it is possible to modulate the physical-chemical gel features by combining different amounts of these crosslinking agents.

*In vitro* experiments with microcapsules gelled with Ca^2+^, Ba^2+^, Ca^2+^-Ba^2+^, and Sr^2+^ have shown further interesting data with regard to the behavior of these specifically formulated gel beads. We observed that all microcapsules, maintained in saline throughout 7 months, shrank regardless of temperature. Additional tests indicated that such behaviour could likely and mainly relate to osmotic factors. In our opinion, the shrinkage observed over time in all kinds of microcapsules can be based on the “Gibbs-Donnan” equilibrium. This is a passive equilibrium, peculiar to semipermeable membranes, that it is set up by negative charges of the alginate's hydrogel carboxyl group not involved in cooperative binding of counterions in the junction zones of the network [17, 28].

Since Na^+^ concentration is very high in the solution around the microcapsules, consequential bead swelling could burst the particles themselves. In contrast, not only did we not observe such swelling but also our particles shrank over time. Through the changes of saline solution, we observed that the gelling ions' concentration rose in the saline solution until an “equilibrium” concentration which maintains the diameters stable over time was reached. The decline in microcapsules' diameter might be due to the interaction of the high-M alginate with the gelling ions in a cooperative phenomenon which strengthens the core more and more over time.

Additionally, the capsules showed elasticity, in terms of their intrinsic ability to adjust their equatorial diameter to the outer environment, from shrinkage to swelling, with no membrane breakage. This, in our belief, relates to the high purity grade of our alginate that lacks interfering substances, such as proteins or endotoxins that may negatively affect the equilibrium by raising negative charges or altering the three-dimensional hydrogel architecture [29]. These findings are consistent with the already reported stress-relaxation mechanism of alginic hydrogels with ionic crosslinks [30]. In this setting, water depletion (inducing capsular diameter shrinkage over time), with subsequent re-establishment of ionic crosslinks (mainly visible between 60 and 120 days for Sr^2+^) is of critical importance.

Furthermore, so far, major obstacles hampering the use of the microcapsules* in vivo* have mainly been related to their poor biocompatibility, leading to posttransplant capsules cellular overgrowth and fibrosis, as well as to their mechanical instability. Here, we showed that all capsule types were long-term stable upon graft into immunoincompetent NOD/SCID mice and proved to be highly biocompatible after graft in immunocompetent CD1 mice. Artificially induced inflammation did not cause any degradation of alginate microcapsules, as shown by the capsules retrieved from CD1 mice pretreated with i.p. LPS. In none of the instances did alginate depolymerization occur, as shown by NMR analysis.

In our study, employment of highly purified alginic acid has unfolded relevance of the counterions, raising the question about which ion or ions combination would be more efficient for encapsulation.

In our opinion, ion selection should be guided by the application which the capsules have been made for. In particular, should the capsules be destined for cell transplantation, a consensus has been reached within the scientific community to employ Ca^2+^ or Ca^2+^-Ba^2+^. While use of Ca-alginate capsules for cell transplant purposes has been sedimented for many years, use of Ba^2+^ has raised more than one objection because of the Ba^2+^ intrinsic toxicity: its LD50 (BaCl) is estimated to range on 1 g/70 kg human [28], and its LDLo (the lowest published lethal dose) is reported to be about 0.8 g [31]. Our microcapsules, gelled with Ba^2+^ (50 mM) or Ca^2+^-Ba^2+^ (25 mM Ba^2+^) and used at a volume of 1 mL, did not induce any acute response in the animals: these results demonstrate that no barium ions were released at toxic levels* in vivo* from our microcapsules and confirm that this cation forms a strong hydrogel with alginate. Moreover, Ba^2+^ concentration assay in the various solutions amounted to 1.2 mM, which is far below the threshold Ba^2+^ levels (5–10 mM) that are known to inhibit K-gated channels. On the other hand, should the capsules be destined as a carrier for drug/molecules delivery system, all cited cations that could be used with care have been taken to check on the membrane's molecular weight cut-off or chemical characteristics of the employed alginates. As far as Sr^2+^ is concerned, recent studies report on the use of this cation for biotechnological and biomedical applications [32]. On a physical-chemical matter, Sr^2+^ holds intermediate properties between Ba^2+^ and Ca^2+^, including affinity to alginate carboxyl radicals. Sr^2+^ is a nontoxic metal showing an* in vivo* behavior similar to Ca^2+^, and can eventually be used in the place of Ca^2+^. Finally, Sr^2+^ is extensively used in pharmacology, and it is part of a number of medical preparations administered to patients (i.e., stronzium ranelate for the treatment of osteoporosis and stronzium lactate to improve diuresis). Moreover, looking at most recent reports on “mechanical memory” and interactions between stem cells and biomaterials [33–35], other parameters could help guide the selection of either the alginic basic polymers (i.e., high-G, high-M, and epimeric), produced by engineered bacteria, or the gelling ions (Ca^2+^, Ba^2+^, Ca^2+^-Ba^2+^, and Sr^2+^), to be considered when stem cells are to be encapsulated, as tools for regenerative medicine and drug delivery. In fact, stem cells are very sensitive to environmental stimuli, and in particular, it seems that a certain capsule type may influence the destiny of the enveloped stems cells (in terms of continuing self-renewal or differentiation into specific cell lineages) [36, 37].

Hence, mechanotransduction applied to the capsule-cell system is to be carefully examined in terms of using the alginate physical-chemical properties to control and eventually influence the stem cell final destination.

## 5. Conclusions

In conclusion, while “standardized” physical-chemical parameters for the preparation of alginate-based microcapsules still are difficult to reach between different laboratories, some proofs of concept and take-home messages may be laid out at this juncture. In order to fabricate homogeneous, stable, and biocompatible alginate microcapsules that are suitable for cell transplant, even for human application, the use of ultrapurified alginate is mandatory. For other purposes, and will strictly depend on, and be guided by the specific application the capsules are destined to, since there is no capsules that are in absolute better than others.

## Figures and Tables

**Table 1:** Ion's concentrations in sodium chloride solution.

	Sample name	Ba (mg/L)	CV	Ca (mg/L)	CV	Sr (mg/L)	CV	Na (mg/L)	CV
Sodium chloride solution after 7 months	CpS Ba	297	1.90*E* − 02	0.3	2.83*E* − 01	<0.05		3095	1.14*E* − 02
	CpS Ca	<0.05		97.2	1.31*E* − 02	<0.05		3115	1.14*E* − 02
	CpS Ca/Ba	167	0.00*E* + 00	67.6	2.20*E* − 02	<0.05		3120	0.00*E* + 00
	CpS Sr	0.3	0.00*E* + 00	2.6	8.32*E* − 02	386.5	2.01*E* − 02	3125	1.58*E* − 02

Sodium chloride solution from “fake saline changes”	CpS Ba	324.5	3.27*E* − 02	0.6	7.07*E* − 01	<0.05		3245	6.54*E* − 03
	CpS Ca	<0.05		100,0	2.48*E* − 02	<0.05		3235	3.28*E* − 02
	CpS Ca/Ba	175	0.00*E* + 00	66.5	0.00*E* + 00	<0.05		3170	0.00*E* + 00
	CpS Sr	0.4	3.54*E* − 01	2.3	3.14*E* − 02	406	0.00*E* + 00	3270	4.32*E* − 03

Sodium chloride solution from saline changes	CpS Ba	129.5	2.73*E* − 02	<0.05		<0.05		3195	2.43*E* − 02
	CpS Ca	<0.05		30.9	6.88*E* − 03	<0.05		3255	2.17*E* − 03
	CpS Ca/Ba	71	9.91*E* − 04	20.8	3.41*E* − 03	<0.05		3230	4.38*E* − 03
	CpS Sr	0.08	0.00*E* + 00	0.7	2.02*E* − 01	103	2.06*E* − 02	3230	2.19*E* − 02

Sodium chloride solution	Blank	<0.05		<0.05		<0.05		3215	1.10*E* − 02
